# Probiotic ice cream using buffalo milk dadih: Microbial, chemical, and sensory characteristics

**DOI:** 10.5455/javar.2025.l888

**Published:** 2025-03-25

**Authors:** Nurzainah Ginting, Yunilas Yunilas, Raden Edhy Mirwandhono, Yuan-Yu Lin

**Affiliations:** 1 Study Program of Animal Science, Faculty of Agriculture, Universitas Sumatera Utara, Medan, Indonesia; 2 Department of Animal Science and Technology, National Taiwan University, Taipei, Taiwan

**Keywords:** Bamboo, colonies, fermentation, ice cream, lactic acid bacteria

## Abstract

**Objective:**

This study aimed to find a quality probiotic ice cream formula by adding dadih, which is a result of the fermentation of buffalo milk in a bamboo tube.

**Materials and Methods:**

This study began with making dadih using 2 types of bamboo (Gigantochloa *verticillata* and *Gigantochloa auriculata Kurz*) to obtain dadih with the highest total probiotic colonies. Dadih with the highest colonies was used to continue the study. Furthermore, dadih was isolated to obtain the dominant lactic acid bacteria (LAB), which was identified molecularly using the 16S rRNA gene with the Polymerase polymerase chain reaction technique. The application of dadih into probiotic ice cream was conducted using a factorial completely randomized design with 2 factors. The first factor was the level of dadih, namely ICD 0 (ice cream without dadih), ICD 1 (5%), ICD 2 (10%), and ICD 3 (15%). The second factor was the length of ice cream storage (week), namely T0 (0), T1 (1), T2 (2) and T3 (3). Examination of pH, protein, fat content, and total bacterial colonies in ice cream was conducted. The research continued with sensory testing with 30 panelists.

**Results:**

The highest total probiotic colonies were 1 × 10^7^ obtained in dadih fermented by *Gigantochloa auriculata Kurz* bamboo dominated by *Weisselia paramesenteroides* strain JCM 9890. pH of dadih was 4.52. Antimicrobial zones of dadih against *Escherichia coli*, *Salmonella,* and *Staphylococcus aureus* were 6.6, 8.1, and 7.7, respectively. The chemical quality test of ice cream showed that the pH was in the range of 6.21–5.61. Protein content ranged from 4.62 to 6.12, while fat content ranged from 6.03 to 8.12. Total ice cream colonies were in the range of 1 × 10^4^ to 2.9 × 10^6^. There was no interaction between the percentage of dadih and the length of ice cream storage time on the parameters of pH, protein, fat content, total LAB colonies, and sensory test result. The higher the dadih concentration, the more acidic the ice cream, and the higher the protein, fat content, and total LAB colonies. The sensory test showed that 15% of dadih was the most preferred by panelists.

**Conclusion:**

The conclusion of this study is that ice cream with a 15% dadih addition is the most preferred and proven probiotic ice cream.

## Introduction

Processed buffalo milk, which is called dali ni horbo, has been consumed by the Batak ethnic community in Indonesia in line with the maintenance of buffalo that has been running for hundreds of years [1]. Most Batak people process buffalo milk with papain enzyme from papaya fruit or bromelain enzyme from pineapple fruit [2]. Meanwhile, people in West Sumatra, Indonesia, also consume processed buffalo milk, which is called dadih, but this dadih is the result of buffalo milk fermentation by utilizing the work of lactic acid bacteria.

The beautiful Samosir Island is one of the tourist destinations in Indonesia. The island, the result of the largest volcanic eruption in Southeast Asia, has the highest buffalo population. The mud buffaloes are the community’s main source of income and are also the animals always used for offerings in traditional ceremonies. Buffalo milk and meat are sources of animal protein for the Batak community, which are processed into culinary delights [3].

The Batak ethnic community believes that consuming dali will keep them healthy, which is their experience of consuming dali. They believe everything that boils down to buffalo will bring good to them because they believe buffalo are among the animals descended from heaven. Scientifically, consuming dali will indeed be healthy. This is related to the nutritional content of buffalo milk [4]. By this time, counseling on processing buffalo milk by fermenting it in bamboo into dadih had reached the Batak community. They are interested because of the healthy bacteria in the dadih, namely probiotics [5].

The content of bacteria found in dadih varies. According to Vargas-Ramella et al. [6], buffaloes raised from different places and different buffalo breeds will carry different cultures of lactic acid bacteria. Research conducted by Pato et al. [7] found lactic acid bacteria (LAB), including* Leu.paramesenteroidesR-8, St. cremorisR-14.* Meanwhile, [8] found *Lactococcus*. LAB bacteria are a challenge to examine because it is believed that these bacteria will bring health to humans.

Samosir Island is currently visited by many foreign tourists. The tourists need for a variety of culinary delights, such as ice cream. However, they must get ice cream that is healthy and nutritious [9]. This encouraged the author to conduct innovation research by making probiotic dadih ice cream. This study was conducted using buffalo milk from Batak, but the process is to ferment buffalo milk using bamboo tubes as done by people in West Sumatra because the result is contained with probiotics. According to Pimentel et al. [10], probiotic ice cream is the most flexible for humans to get healthy ice cream, while ice cream is one of the preferred foods for all age groups of humans.

## Materials and Methods

### Ethical approval

Research involving human participants in this study does not violate their welfare. The materials used are buffalo milk that had just been milked from buffalo farming, two species of bamboo (*Gigantochloa* *verticillata*/Gombong bamboo/green color and *Gigantochloa auriculata Kurz*/Kuning bamboo/yellow bamboo stalks), dadih, which was prepared for this study, MRSA media (Merck-Germany), MRSB (Merck-Germany) to identify LAB, SSA (Oxoid UK) to conduct antimicrobial tests, *Escherichia coli* ATCC^®^ 8739, *Salmonella enterica* serotype (ser.), *and Staphylococcus aureus *isolates as antimicrobial test materials, and ice cream-making ingredients.

The tools needed are Petri dishes for the LAB isolation process, pans for cooking ice cream ingredients, freezers for compacting ice cream dough, and mixers for stirring ice cream dough that has solidified. This study involves human participants; however, it does not violate the welfare of humans.

### Preparation of dadih

Filtering fresh buffalo milk so that it is clear of impurities brought in during buffalo milking. Pasteurizing the milk by heating it on medium heat to a temperature of 65°C. Then cool the milk to 37°C and put the milk into bamboo segments. Covering the bamboo-segment with banana leaves that have been wilted by roasting them over a fire. Storing the bamboo in a dry room for 2 days. After 2 days, dadih is ready to be used to make probiotic ice cream.

The making of dadih in this study follows what has been done by the people of West Sumatra. They process milk into dadih because fermented milk has a longer shelf life than that without fermentation. Amelia et al. [11] mentioned that in the process of making dadih, lactic acid is formed by lactic acid bacteria in the bamboo tube. The atmosphere of the dadih becomes acidic, protecting the dadih from spoilage bacteria while extending the shelf life.

Dadih contains 73.55% water, 7.96% protein, 5.53% fat, and pH 5.16 [12]. There are also amino acids (13 essential amino acids and three nonessential amino acids), so it can be a nutritious food. pH around 4.0 makes dadih not favored by contaminant microbes [13].

### Total plate count calculation of dadih

The TPC method was performed on dadih from both green bamboo and yellow bamboo. Before microorganisms are grown in the media, a dilution of the sample using the physiological solution is first carried out. Dilutions were made from 10⁻³ to 10⁻⁶.

### LAB isolation

Milk that had been fermented with bamboo and became dadih was diluted to a dilution of 10–6 using MRSB (Medium deMan Rogosa Sharpe Broth) media. Microbes were grown on MRSA (Medium deMan Rogosa Sharpe Agar) media using dilutions of 10⁻⁴ to 10⁻⁶ using the pouring cup method. Counted microbial colonies that grew at dilutions of 10⁻⁴ to 10⁻⁶ using a colony counter. Observed differences in microbial colony morphology, including shape, margin, elevation, size, texture, and color. Any microbes that had prominent morphological differences were purified on different MRSA media. In this study, the two most prominent colonies were found and then purified to continue with the test molecularly, 16S rRNA.

### LAB molecular identification using 16S rRNA

The aim was to identify the bacteria molecularly, which was done by Indolab, Singapore.

### Antimicrobial test

This test was conducted to determine the antimicrobial ability of dadih. The production of antimicrobial compounds from lactic acid bacteria was carried out by entering 10 ml (concentration equivalent to McFarland 0.5 solution) into 100 ml of MRSB media and then incubated at 280C for 7 days and shaken with an *orbital shaker* at 110 rpm. Isolation of antimicrobial compounds is done by inserting into the bacterial culture as much as 10 ml of ethyl acetate solution and shaking for 10 min, then allowing it to stand until two layers of liquid are formed. The addition of ethyl acetate was repeated 2 times. The solution of ethyl acetate extraction was then evaporated to form a dry extract.

The dry ethyl acetate extract was then dissolved with *dimethyl sulfoxide* (DMSO) in a ratio of 1:1 (weight of dry extract: DMSO). The concentration of the extract in DMSO solvent was 50%. The antimicrobial test was carried out by the Kirby-Bauer method. A total of 30 µl of the extract was dripped on sterile disc paper that had been placed on media that had been inoculated with pathogenic bacteria. The zone of inhibition formed was then measured using a caliper.

### Manufacture of probiotic ice cream

The process of making ice cream is done by preparing the tools and materials needed and weighing all the ingredients according to the treatment. Coconut milk, sweetened condensed milk, cornstarch, sugar, plain water, and salt cooked until boiled. Next, let it cool down until lukewarm and add dadih. When it has cooled down, put it in the refrigerator to increase the viscosity of the dough. After the dough has hardened, mix the dough until soft and fluffy while adding emulsifier and pure mango. Then store in the freezer for 24 h. These physical treatments result in air bubbles, fat globules, and ice crystals. Making this ice cream is done in the research of [14] that ice cream dough containing protein, sugar, salt, and polysaccharides causes the ice cream dough to become concentrated, which is then frozen. Ice cream is made with the addition of yellow bamboo dadih, which has the most LAB colonies.

Because the ice cream in this study is probiotic ice cream, a TPC test was conducted. 1 gm sample of ice cream was taken aseptically and then diluted in distilled water from 103 to 106. Ice cream TPC counts were conducted weekly for three consecutive weeks because it was assumed that the ice cream would run out in 3 weeks.

### Sensory test of ice cream

The sensory test was assisted by 30 untrained panelists [15] consisting of students, faculty employees, and lecturers. There were 17 females and 13 males with an age range between 20 and 55 years old.

Sensory test including flavor, texture, and aroma of ice cream. Flavor consists of 7 scores, namely, 7 = Very good, 6 = Good, 5 = Somewhat good, 4 = Neutral, 3 = Somewhat bad, 2 = Not good, and 1 = Very bad. Texture consists of 7 scores, namely, 7 = very fine, 6 = fine. 5 = Somewhat fine, 4 = Neutral, 3 = Somewhat coarse, 2 = Coarse, 1 = Very coarse. The aroma in the food industry is considered important because it can quickly provide the results of the assessment of the product about whether or not a product is accepted. The test for aroma consists of 7 scores, namely, 7= Very like, 6= Like, 5= Somewhat like, 4= Neutral, 3= Somewhat dislike, 2= Dislike, and 1= Very dislike.

## Results and Discussion

### Preparation of dadih

In this study, dadih was made using two species of bamboo that can easily be found on Samosir Island, namely green bamboo with the local name Gombong bamboo (*Gigantochloa auricullata*) and yellow bamboo with the local name Kuning bamboo (*Gigantochloa auriculata Kurz*). The results of making dadih obtained solid part/dadih and liquid part/whey. The colony count of lactic acid bacteria in both dadih and whey was carried out with the results as listed in Table 1.

Dadih can be categorized as a probiotic. Probiotics are defined as live microorganisms that have health effects on the host. Various clinical effects have been found in humans, including not only improving fecal consistency and the balance of gut-dwelling bacteria but also reducing allergies and preventing influenza infection [16].

In this study, dadih was in an acidic atmosphere with a pH of dadih around 4. LAB dadih fermented buffalo milk, which caused the milk to clump into a semi-solid, yellowish-white form. In addition, dadih is sour due to the production of organic acids, especially lactic acid from lactose fermentation. Dadih has a distinctive aroma from the combination of bamboo powder and volatile compounds. The result of this fermentation includes water so that two layers are formed, namely dadih and liquid. The dadih floats at the top, and the liquid is called whey at the bottom [17].

The more the LAB total colonies, the more lactose is metabolized, and the more organic acids are produced so that the pH becomes more acidic. In Table 1, it can be seen that the total colonies are higher in dadih fermented on *Gigantochloa auriculata Kurz* bamboo, so the pH of the dadih is more acidic.

**Table 1.** pH and Total LAB Colonies of dadih and whey.

Bamboo Species	pH	Total LAB Colonies	Total LAB Colonies
		Solid part (Dadih)	Liquid part (Whey)
Gigantochloa verticillata	4.63	5 × 10^6^	2 × 10^6^
Gigantochloa auriculata Kurz	4.52	1 × 10^7^	3 × 10^6^

Source : primary data.

**Table 2.** Sequencing primer name of dadih LAB isolate.

Sequencing Primer Name Primer Sequences	PCR Primer Name Primer Sequences
785F 5' (GGA TTA GAT ACC CTG GTA) 3'	27F 5' (AGA GTT TGA TCM TGG CTC AG) 3'
907R 5' (CCG TCA ATT CMT TTR AGT TT) 3'	1492R 5' (TAC GGY TAC CTT GTT ACG ACT T) 3'

Source : Primary data.

### Total plate count calculation of dadih

TPC was calculated on dadih from Gombong bamboo (*Gigantochloa verticillata*) and Kuning bamboo/yellow (*Gigantochloa auriculata* Kurz). The LAB colonies are shown in Table 1. There were more colonies in yellow bamboo than in green bamboo. The higher total colonies may be supported by the characteristics of yellow bamboo. Yellow bamboo is high in carbohydrates and also contains sugar.

Yellow bamboo is used for medicinal purposes, which is related to the secondary metabolites it contains. Microbes need sugar, which is an indication of more simple carbohydrate availability, to thrive [18].

### LAB isolation

Furthermore, because there were more colonies in yellow bamboo, the focus of the research was on yellow bamboo. In this study, we isolated two dominant gram-positive bacteria and further identified them using the PCR method.

### Identification of lab using 16S rRNA with PCR

Gram-positive bacteria that have been found were purified and molecularly identified using the 16S rRNA gene-specific primers (Table 2) for the polymerase chain reaction (PCR) technique. The result states that both isolates are the same strain. By using 16S rRNA gene sequencing, both isolates were included in the genus Lactobacillaceae and registered to GenBank as *Weissella paramesenteroides* strain JCM 9890. According to the taxonomy database at the National Center for Biotechnology Information, *Weissella* consists of 22 species, including *Weissella* *paramesenteroides*. It is a lactic acid bacteria that has probiotic potential [30].

### Antimicrobial test

Antimicrobial tests of dadih against pathogenic bacteria (*Escherichia coli* ATCC^®^ 8739, *Salmonella enterica* serotype (ser.) *Typhimurium* ATCC^®^ 14028, and *Staphylococcus aureus*) were conducted. Dadih was filtered, and then its extract was centrifuged at 3000 rpm, 4°C for 10 min. The supernatant obtained was collected and used for antimicrobial tests against pathogenic bacteria. The results are shown in Table 3.

The results of the antimicrobial test are reflected through the inhibition zone (mm). According to Ismail et al. [20], there were three categories of inhibition or antimicrobial zone, i.e., very strong if the inhibition zone >20 mm, strong 10–20 mm, medium 5–10 mm, and weak <5 mm. Dadih has a medium power to suppress the development of *Salmonella*, *E. coli,* and *S. aureus*. The ability of dadih to suppress the growth of pathogenic bacteria indicates that dadih is a probiotic.

Probiotics are defined as live microorganisms that, when administered in adequate amounts, confer a health benefit on the host [16]. The dadih in this study consisted of probiotic lactic acid bacteria Weissella paramesenteroides strain JCM 9890. As mentioned earlier, people on Samosir Island feel the benefits of dadih consumption for their health. This phenomenon is by the results of research by Gunathunga et al. [21] that buffalo milk dadih is a rich source of lactic acid bacteria (LAB) and has a broad range of probiotic characteristics.

### Manufacture of probiotic ice cream

According to Genovese et al. [9], consumers want ice cream that is tasty yet healthy. In addition, consumers want ice cream that has excellent texture quality and flavor. Therefore, it is time-sensitive to produce ice cream that is nutritionally beneficial, without reducing the pleasure of consumption. The integration of functional ingredients with the most suitable formula should enhance the ice cream’s quality.

The preparation of probiotic ice cream in this study is different from that conducted by Akalin et al. [22]. They added a starter culture of *L. acidophilus* and *B. lactis* Bb12 to the dough (108 cfu/gm), mixed well, and fermented for approximately 3.5 h at 40°C until the pH of 5.5 was reached. Therefore, only *L. acidophilus* and *B. lactis* Bb12 cultures were found in their ice cream. While the ice cream in this study added dadih, various probiotic bacteria were found, including *L. lactis*, *L. plantarum,* and *Weissella paramesenteroides*. This study utilized naturally developed cultures in bamboo tubes. Therefore, through the TPC test of lactic acid bacteria, total colonies were found to be significantly different for each dadih addition treatment of 5%, 10%, and 15% (Table 6).

The nutritional quality of ice cream in this study is still within the quality standards set by the Indonesian National Standard (Table 5). There was no interaction between the percentage of dadih and the length of ice cream storage time on the parameters of pH, protein, fat content, and total LAB colonies. The pH of dadih ice cream from the first to the third week experienced a slight increase, meaning that the pH of the ice cream became slightly more acidic, indicating that the probiotic bacteria were still working. This is by the study of [10] that probiotics survive even though they are stored for a long time in frozen conditions. In ice cream, lactic acid bacteria break down lactose into lactic acid. With the increasing number of lactic acid bacteria that use lactose, the more sugar sources that can be metabolized, the more organic acids are produced so that the pH is also lower. The pH of the ice cream became more acidic (p < 0.05) along with an increasing percentage of dadih. Dadih in this study has a pH of 4.63, while research by Bahow et al. [24] found that probiotic ice cream with the addition of starter *Streptococcus thermophilus* and *Lactobacillus acidophilus* (3%, 6%, and 9%) achieved a pH of 5.35, 5.32, and 5.24, respectively. Normal ice cream dough has a pH value of 6.67. In this study, the pH of ice cream without the addition of dadih was 6.21. The use of coconut milk, which is acidic, may have contributed to this study’s lower pH.

**Table 3.** Antimicrobial activity of dadih’s LAB against *Escherichia* *coli*, *Salmonella*, *Staphylococcus aureus.*

Dadih’s LAB	Inhibition zone (mm)
*Escherichia coli*	6.6
*Salmonella*	8.1
*Staphyllococcus aureus*	7.7

Source : Primary data.

**Table 4.** Ice Cream formulation with buffalo milk dadih addition.

	ICD 0	ICD 1	ICD 2	ICD 3
Dadih (gm)	0	150	300	450
Coconut milk (gm)	1,130	1,130	1,130	1,130
Sweetened condensed milk (gm)	152	152	152	152
Cornstarch (gm)	260	260	260	260
Sugar (gm)	500	500	500	500
Plain water (gm)	450	450	450	450
Emulsifier (gm)	6	6	6	6
Salt (gm)	20	20	20	20
Pure manggo (%)	300	300	300	300

The percentage of protein was found to increase as the percentage of dadih increased (*p *< 0.05). The addition of 15% dadih resulted in ice cream containing a high protein concentration. Dadih itself contains high protein as it comes from buffalo milk. In addition, LAB contains amino acids that increase the protein of ice cream. However, what is more influential on the protein content of ice cream is the protein content in dadih, and this is reinforced by the statement of Arslaner and Salik [14] that in the condition of ice cream, nutritive constituents such as protein are more influenced by the initial conditions of ice cream.

Fat concentration is higher than the minimum SNI standard. This is because coconut milk was used in this study. Research by Fuangpaiboon and Kijroongrojana [25] found that fat from coconut milk is an important ingredient in ice cream as it provides the desired flavor, softness, elasticity, and emulsification, although coconut-derived fat is not good for obese people due to its 8%–12% fat content. Begum et al. [26] mentioned that non-dairy formulations of coconut milk were preferred over dairy products in terms of taste and aroma during the sensory test. They found crude fat of coconut milk ice cream was 10.52%–11.62%, while in this study it was 6.03%–8.12%. After becoming dadih, the fat content increased. The fat content in dadih is influenced by the fermentation of lactose into lactic acid, which causes casein clumping followed by fat clumping. This is consistent with the research of Hanani et al. [27], who found the fat of buffalo milk to be around 6.17% and, after becoming dadih, 7.34%.

**Table 5.** Ice cream quality requirements (Indonesian National Standard, 1995) [23].

Criteria	Requirements (minimum)
Fat (% b/b)	5,00
Sugar (% b/b)	8,00
Protein (% b/b)	2,70
Total solids (% b/b)	3,40

**Table 6.** Chemical and biological quality of probiotic ice cream.

Treatments	TPC (CFU/gm/week)			
	0	1	2	3
ICD 0	0	0	0	0a
ICD 1	5 × 10^4^	3 × 10^4^	2 × 10^4^	1 × 10^4^b
ICD 2	7 × 10^5^	5 × 10^5^	3 × 10^5^	3 × 10^5^c
ICD 3	2.9 × 10^6^	2.6 × 10^6^	2.6 × 10^6^	2.5 × 10^6^d
	pH/week			
	0	1	2	3
ICD 0	6.21	6.20	6.20	6.20d
ICD 1	5.87	5.86	5.81	5.80c
ICD 2	5.71	5.70	5.67	5.66b
ICD 3	5.64	5.65	5.62	5.61a
	Protein/%/week			
	0	1	2	3
ICD 0	4.62	4.62	4.62	4.62a
ICD 1	5.72	5.72	5.73	5.73b
ICD 2	5.98	5.98	6.00	6.00c
ICD 3	6.11	6.11	6.12	6.12d
	Fat/%/week			
	0	1	2	3
ICD 0	6.03	6.03	6.03	6.03a
ICD 1	6.98	6.98	6.98	6.98b
ICD 2	7.55	7.55	7.55	7.55c
ICD 3	8.12	8.12	8.12	8.12d

a-c Means with different letters in the same column are significantly different (*p* < 0.05)

Source: Primary data.

### Sensory characteristics of ice cream

The sensory test was conducted by 30 untrained panelists consisting of students, employees, and lecturers. Sensory testing was carried out on the aroma, texture, and taste-flavor of ice cream. There was no interaction between the percentage of dadih and the length of ice cream storage time on sensory characteristics of ice cream (Table 7). Referring to Table 4, the composition of the ice cream formula in each treatment is the same; the only difference is the dadih concentration. Panelists said that the aroma of treatment without dadih with dadih was different. The treatment with dadih was preferred because there was a fresh sour aroma. In research conducted by Rifdi et al. [28] from 20 panelists, 9 panelists liked and 11 panelists liked the dadih ice cream.

The texture of dadih ice cream with the addition of 10% and 15% dadih was preferred by the panelists. Fat content affects the texture of ice cream because fat helps provide texture density in ice cream [26]. The higher the fat content of ice cream, the softer the ice crystals formed. The addition of Dadih levels of 10% and 15% causes the fat content of ice cream to be 7.55% and 8.12%, respectively. The same thing also happened in the research by Hanani et al. [27], who found a soft ice cream with fat content of about 7.34.

The addition of 15% dadih gives a taste that is highly preferred by panelists because the ice cream tastes fresher and like it contains cheese. This is influenced by the cheese-like flavor of buffalo milk dadih. The dadih flavor is like fresh cheese: fresh, delicious, and milky. In this study, the most liked dadih ice cream was with a pH of 5.61, while a study by Ambri et al. [29] found that the most preferred dadih ice cream among panelists was a fresh, sour taste with a pH of 5.07. In addition, Rifdi et al. [28] mentioned that the favorability test is the most important factor in knowing how consumers accept a product.

**Table 7.** Mean sensory value of probiotic ice cream with dadih addition.

Treatments	Aroma			
	0 (week)	1 (week)	2 (week)	3 (week)
ICD 0	5.8	5.8	5.8	5.8a
ICD 1	6.4	6.4	6.4	6.4b
ICD 2	6.4	6.4	6.4	6.4b
ICD 3	6.9	6.9	6.9	6.9c
	Texture			
	0 (week)	1 (week)	2 (week)	3 (week)
ICD 0	5.8	5.8	5.8	5.8a
ICD 1	6.3	6.3	6.3	6.3^b^
ICD 2	6.3	6.3	6.3	6.3^b^
ICD 3	6.3	6.3	6.3	6.3^b^
	Taste-flavour			
	0 (week)	1 (week)	2 (week)	3 (week)
ICD 0	5.7	5.7	5.7	5.7a
ICD 1	6.3	6.3	6.3	6.3b
ICD 2	6.5	6.5	6.5	6.5b
ICD 3	7.2	7.2	7.2	7.2c

a–c Means with different letters in the same column are significantly different (*p* < 0.05) Source: Primary data.

## Conclusion

In this study, it was found that dadih contains LAB colonies 1 × 10^7^ with pH 4.52, which was fermented using bamboo *Gigantochloa auriculata Kurz*. Based on rRNA analysis, the colony that dominated the dadih was *Weisselia paramesenteroides* strain JCM 9890. The antimicrobial zones of dadih against *Escherechia coli*, *Salmonella,* and *S. aureus* were 6.6, 8.1, and 7.7, respectively. The chemical quality test of ice cream indicated that the pH was in the range of 6.21–5.61. Protein content ranged from 4.62 to 6.12, while fat content ranged from 6.03 to 8.12. Total ice cream colonies were in the range of 1 × 10^4^ to 2.9 × 10^6^. There was no interaction between the percentage of dadih and the duration of ice cream storage on the parameters of pH, protein, fat content, total LAB colonies, and sensory test result. The higher the dadih concentration, the more acidic the ice cream, the higher the protein and fat content, and the higher the total LAB colonies. The sensory test showed that 15% dadih addition was the most preferred by panelists. This study concludes that ice cream with a 15% dadih addition is the most preferred and proven probiotic ice cream.

## List of abbreviations

LAB, lactic acid bacteria; MRSA, de man rogosa and sharpe agar; MRSB, de man rogosa and sharpe broth; NCBI, The National Center for Biotechnology Information; PCR, polymerase chain reactions; SSA, salmonella shigella agar; SNI Indonesian national standard; TPC, total plate count.
